# Gene doping in horse racing and equine sports: Current landscape and future perspectives

**DOI:** 10.1111/evj.14418

**Published:** 2024-09-12

**Authors:** Maria Puchalska, Olga Witkowska‐Piłaszewicz

**Affiliations:** ^1^ Department of Large Animals Diseases and Clinic, Institute of Veterinary Medicine Warsaw University of Life Sciences Warsaw Poland

**Keywords:** doping control, gene doping, horse, racehorses, transgenes, welfare

## Abstract

Gene doping, the use of gene therapy or genetic manipulation to enhance athletic performance, has emerged as a potential threat to the integrity and welfare of equine sports, such as horse racing and equestrian sports. This review aims to provide an overview of gene doping in horses, including the underlying technologies, potential applications, detection methods, ethical concerns and future perspectives. By understanding the current landscape of gene doping in horses, stakeholders can work together to develop strategies to safeguard the integrity of equine sports.

## 1 INTRODUCTION

Gene doping and misuse of gene therapy has emerged as a potential threat to sports performance and although, in human sports, there has been no registered abuse of this kind of doping, there is a recognition of the possible danger. In 2004, the World Anti‐Doping Agency (WADA) banned gene doping in humans and animals, and since then, the search for accurate ways of detecting gene doping has progressed.^1^ WADA describes gene‐doping as: ‘The use of nucleic acids or nucleic acid analogs that may alter genome sequences and/or alter gene expression by any mechanism. This includes but is not limited to gene editing, gene silencing, and gene transfer technologies’.^2^ Gene doping is not only a danger to the possible falsification of sports performance but also to athletes' health and raises concerns for human sports as well as for sports involving animals. The International Federation of Horseracing Authorities (IFHA) and the International Federation for Equestrian Sports (FEI)^3^ have prohibited gene doping. In this article, the potential of gene doping usage in horse athletes, especially in the racing industry, is reviewed.

It is worth mentioning that horses are serving as a model for research in gene therapy for humans.^4^, ^5^ Due to their longevity and bigger anatomical features like joints, they are a suitable animal study model for developing such treatments. The field is also relevant to the health of equine athletes^6^ and the characterisation of the horse genome sequence in 2006 has allowed for better scanning of the genomes.^7^ These findings provided a better understanding of certain genes linked to the sports performance of the horses. Gene therapy is still a developing field and recent studies are showing promising beneficial effects^6^, ^7^; however, its use creates space for the illegal use of gene therapy to enhance the performance of sport horses and it is forbidden to treat equine athletes.^6^ Motives for misuse of gene therapy may be financial and, as an example in Great Britain, horse racing has generated £3.5 billion annual expenditure.^8^ Gene doping also raises concerns for the welfare of equine athletes. There is a danger that it could be used to hide ailing horses, leading to more serious injuries and threats to their health, and there is also the possibility of harmful side effects. Another menace is the fact that developing gene doping in horses may facilitate the transfer of these techniques for use in humans.

## 2 GENE THERAPY TECHNIQUES

**FIGURE 1:**
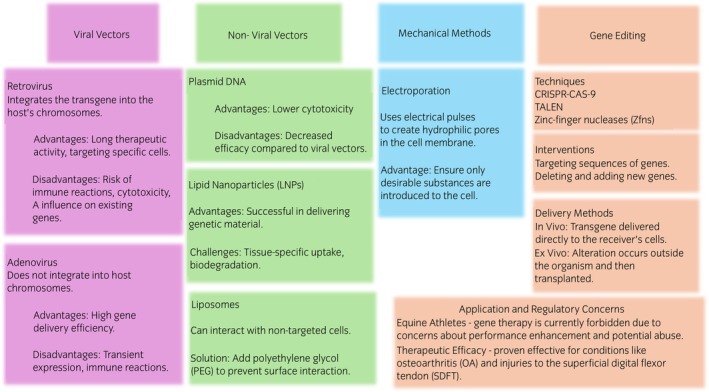
Summary of various gene therapy techniques, including viral and non‐viral vectors, mechanical methods and gene editing approaches.

Gene therapy is a promising treatment in some diseases as it can modulate gene expression and modify genes. One of the methods is injecting a gene (transgene) in a ‘messenger’, for example, in a viral vector or plasmid.^9^ Possible methods have been summarised in Figure 1. The most common vectors are viruses, as they introduce the carried gene to the genome of the receiver. Retroviruses provide the mechanism to allow the transfer of gene(s) into chromosomes, whereas adenoviruses do not. Some of the advantages, depending on the type of chosen viral vector, are long therapeutic activity, targeting specific cells, and significant capacity. They may differ throughout the viruses. Sometimes the introduction of a viral carrier poses an unwanted immune reaction, cytotoxicity or a danger of influencing the genes that exist already in the genome. The other option is the usage of non‐viral transporters, such as plasmid DNA or chemicals like a lipid in the form of a liposome.^10^, ^11^, ^12^ They are characterised by lower cytotoxicity but also decreased efficacy. However, in recent years, the efficacy of lipid nanoparticles has been evaluated. They are successful in delivering the desired genetic material but present some challenges, for instance, tissue‐specific uptake or biodegradation.^13^, ^14^, ^15^ One of the concerns for chemical transporters is that they can interact with the non‐targeted cells of the organism; for instance, liposomes react with lipoproteins. There are ways to overcome this, such as adding polyethylene glycol, which prevents the interaction of the liposome's surface.^16^ There is also a possibility of mechanical methods such as electroporation, that rely on electrical pulses delivered to the cell that create hydrophilic pores in the cell membrane. This method ensures that only desirable substances will be introduced to the cell.^17^ The overall goal of introducing a foreign gene is often the production of a designated protein.^12^

Secondly, gene intervention can take place by gene editing [Clustered Regularly‐Interspaced Short Palindromic Repeats (CRISPR–Cas9), Transcription activator‐like effector nucleases (TALEN) or zinc‐finger nucleases (ZFNs)], which is a more advanced technique that assumes changing genetically an embryo or an egg to receive desired modified offspring.^6^, ^18^ The intervention can include targeting sequences of genes, and deleting and adding new genes and this kind of alteration will be passed on to the progeny. The modifications can be delivered to the organism in vivo or ex vivo. In vivo, the transgene is delivered to the receiver's cell, where it is expressed. In ex vivo, the alteration occurs outside of the target organism and the outcome product is transplanted to the desired place of transgene expression in the organism of the receiver.^9^

Currently, although gene therapy of equine athletes is forbidden by regulatory bodies^6^, ^19^ the therapeutic efficacy of gene therapy has been proven for OA and injuries of the superficial digital flexor tendon (SDFT).^20^ In the majority of the studies that used gene delivery methods, the route of administration was intra‐articular,^4^, ^6^, ^10^ at the specific injury place,^6^, ^20^ with some studies having also used intramuscular injections.^21^, ^22^, ^23^

## 3 POTENTIAL APPLICATIONS OF GENE DOPING IN HORSES

### 3.1 The possible genes that could influence horses' performance

#### 3.1.1 Exercise adaptation genes

Research has identified numerous genes involved in exercise adaptation by examining global mRNA expression in skeletal muscle post‐treadmill training. Biopsy samples obtained from *musculus gluteus medius* taken 4 h after the training showed increased expression of 932 genes. These genes primarily influence the actin cytoskeleton, affecting the mitogen‐activated protein kinase pathway responsible for cell proliferation and differentiation^7^ and they impact insulin signalling and mTOR pathways that also affect cell proliferation.^24^, ^25^ All these mechanisms contribute to muscle adaptation to the effort.^26^

#### 3.1.2 Muscle mass genes

Genes that impact muscles' mass, an indicator of an athlete's capability, are a possible target for manipulation, such as the myostatin gene (*MSTN*), the expression of which has a significant effect on the mass of the skeletal muscles. By autocrine or paracrine signalling it inhibits the growth of skeletal muscles and restrains the number and dimension of muscle fibres.^27^ Blocking of this gene could be a potential target in gene doping, as it would enhance muscle growth. Furthermore, research has indicated that variants within the *MSTN* gene are responsible for a preferred racing distance for elite racehorses.^28^ In a recent study, Moro et al.^29^ achieved a knockout MSTN horse embryo by the usage of somatic cell nuclear transfer and CRISPR/Cas9. In addition, glycoprotein follistatin, which inhibits the expression of MSTN, has been proven in mice and macaques to promote the development of muscles. Whether follistatin gene manipulation would upgrade race performance is unknown, but there has been research in human competitors showing the performance enhancers anabolic steroids increase follistatin concentration.^7^

#### 3.1.3 Insulin‐like growth factor‐1

Insulin‐like growth factor‐1 (IGF‐1) influences muscle mass and strength. It not only inhibits MSTN, but also promotes cell growth and protein synthesis. In mice, gene therapy with IGF‐1 caused around 15% growth in muscle mass without any additional training. When combined with training, it reached about 30%. Stopping the training revealed slower muscle loss,^30^ which might be relevant to avoid loss of muscle mass decreasing in equine athletes experiencing a break in training. Another study showed that injecting IGF‐1 affects only targeted muscles, which allows the prevention of side effects.^7^ It has been suggested that IGF‐1, interleukin‐1 receptor antagonist (IL‐1ra) and matrix metalloproteinase 2 (MMP2) are suitable biomarkers for early detection of horses at risk of injuries^31^; however, only basal values of IGF‐1 have value as markers of fitness status in racehorses.^32^

#### 3.1.4 Muscle contraction genes

The *ACTN3* gene, which encodes the sarcomeric protein α‐actinin‐3, is crucial for rapid muscle contraction in type II myofibres. It also influences the adaption to exercise and glycogen metabolic paths in the muscles.^33^ There are polymorphisms in *ACTN3* gene, for example in humans one single‐nucleotide polymorphism (SNPs) is believed to divide the endurance capabilities of an athlete from the highest‐performing sprinters.^34^ At this moment, such SNPs have not been identified in horses.^7^ However, there is a study that has localised SNPs that may impact athletic performance.^35^ Therefore, with the evolution of genetic techniques, this could be a target of future doping abuse, as well as a biomarker of performance potential.^33^

#### 3.1.5 Growth hormone

Somatropin [growth hormone (GH)], which stimulates protein synthesis and muscle growth, has been abused in human sports. Although recent studies have proven that its use in healthy individuals does not significantly improve sports performance, it could also be a target for gene doping because of its well‐known benefits. In 1992, a recombinant form of equine GH (reGH) was proposed to improve nitrogen balance in aged horses and it showed no impact on sports performance. Methods of detecting the usage of these drugs are well‐developed and there have been no reports of their abuse.^7^

#### 3.1.6 Oxygen transport and erythropoiesis genes

High maximal aerobic capacity, which enhances the oxygen flow in the body, influences athletic performance.^36^ This includes the transport of oxygen in the blood by haemoglobin when the tissue demand rises during exercise.^25^ Blood oxygen transport capacity is a potential target for gene doping. In the past, there were reports of the misuse of erythropoietin (EPO) in human athletes and it, and related recombinants like darbepoetin, are banned in sport. EPO is produced by the peritubular cells of the kidneys and stimulates erythropoiesis by binding with receptors located in the bone marrow.^37^ A vector plasmid with EPO was introduced to the public in 2003 but very quickly withdrawn. There were no confirmed reports of its abuse in equine sports and it would probably cause an immunological response in horses.^7^ However, genetic manipulation of EPO remains a possibility.

Hypoxia‐inducible factor 1 (HIF‐1) takes part in the expression of genes responsible for not only erythropoiesis, but also angiogenesis, mitochondrial biogenesis and glucose metabolism. This makes it a perfect target for gene doping as all these processes influence athletic capability.^38^ For instance, the more mitochondria there are in muscle, the greater the muscle oxygen usage.^25^ Hypoxia induces the expression of HIF‐1, which upregulates vascular endothelial growth factor (VEGF) transcription.^7^ Behairy et al.^19^ showed a significant increase in the expression of VEGF after exercise. VEGF also promotes angiogenesis, vasculogenesis and endothelial cell growth, contributing to physical performance adaptation.

#### 3.1.7 Performance‐related polymorphisms

Literature suggests that polymorphisms of certain genes could impact performance abilities, including CK (creatine kinase, muscle), COX4I2 (cytochrome c oxidase, subunit 4, isoform 2), with strong evidence for a PDK4 (pyruvate dehydrogenase kinase, isoenzyme 4) gene.^39^ COX4 takes part in mitochondrial respiration and ATP production. The expression of COX4 rises in equine skeletal muscles during physical exercise and positively impacts performance. CK plays a role in the phosphorylation paths of ATP and certain phosphagens. An experiment on CK‐knockout mice showed that it improved their resistance to exhaustion.^7^ Expression of PDK4 influences glucose metabolism and regulates oxidation of fatty acids that are used in highly efficient generation of ATP (β‐oxidation) and Hill et al.^40^ showed associations between two SNPs within the *PDK4* gene and race performance.

#### 3.1.8 Peroxisome proliferator‐activated receptors

Genes that encode the peroxisome proliferator‐activated receptors (PPARs) appear to have a major role in exercise capacity, as PPARs participate in the regulation of the genes that impact fatty acids oxidation and glucose metabolism.^7^, ^41^ There have been reports supporting the angiogenesis‐inducing effects of PPAR‐α acting synergically with VEGFB and PPAR delta is proposed as a marker of potential endurance in horses, considering its increased expression during exercise.^42^, ^43^

### 3.2 Phenotype modification

The desired racehorse phenotype is well established through the years of breeding and in Thoroughbred horses speed is the trait that is particularly treasured. Studies have tracked quantitative trait loci that modulate characteristics like weight and height^12^, ^44^ and, as mentioned above, the *MSTN* gene influences muscle mass. Rooney et al.^45^ conducted a study which showed that the insertion of short interspersed nuclear elements (SINE) decreases MSTN expression, consequently influencing muscle development. Another gene that could be the target of gene doping abuse is TBX15 (T‐box transcription factor), which not only influences skeletal development, but also skeletal muscle fibre differentiation. Some sources indicate a connection between poor conformation and susceptibility to possible injuries.^37^, ^46^

Several studies have suggested that the coat colour does not influence racehorse performance.^47^, ^48^ However, there are scientific reports of some gene loci responsible for coat colour that take part in some pathological conditions. For instance, in lavender foal syndrome in Arabian foals, there is a coat colour dilution l that is associated with a lethal deletion mutation in the *myosin 5A* gene.^49^

### 3.3 Influencing disease susceptibility

Gene doping has the potential to reduce disease susceptibility with a range of possible target genes. Thorpe et al.^50^ has shown that injury of the SDFT involves microdamage and the enhanced expression of the *MMP‐1* gene (or collagenase‐1). Increased expression *MMP‐1* gene is also noted in OA, which is also common among racehorses.^51^, ^52^, ^53^ A study by Ruan et al.^54^ on a mouse OA model shows that gene therapy with an adenoviral vector that expresses proteoglycan 4 (PRG4) can prevent the evolution and development of OA in older individuals. IGF‐1 influences proteoglycan synthesis and enhancement in the type II collagen creation^55^ while interleukin 1 receptor antagonist (IL1RN encodes the IL‐1ra). IL‐1ra acts as an inhibitor of interleukin‐1 and has been used as an anti‐inflammatory agent in joint diseases^56^ with the potential for treating clinical signs and modifying the progression of trauma‐induced OA.^57^ In one clinical study, the plasmid DNA encoding two therapeutic species‐specific growth factors VEGF164 and fibroblast growth factor 2 (FGF2), were successfully used to treat SDF tendinitis and desmitis of suspensory ligament branch.^58^

Possible targets for gene therapy are summarised in Table 1.

**TABLE 1:** Potential applications of gene doping in horses.

Potential applications of gene doping in horses	Gene	Action	Possible effects
Influencing horses' performance	MSTN	Inhibiting the growth of skeletal muscles	Promoting muscle growth by blocking expression
	IGF‐1	Inhibiting MSTN Promoting cell growth and protein synthesis	Growth in muscle mass Slower muscle loss
	ACTN3	Production of a sarcomeric protein α‐actinin‐3	Enhancing the adaption to the exercises
	GH	Stimulating protein synthesis and muscle growth	Muscle growth
	EPO	Stimulating the erythropoiesis	Increasing endurance, effectiveness of the performance
	HIF‐1	Expression of genes responsible for erythropoiesis, angiogenesis, mitochondrial biogenesis, glucose metabolism	Promoting athletic capability
	VEGF	Promoting of the angiogenesis, vasculogenesis, endothelial cell growth	Increasing physical performance adaption
	COX4I2	taking part in the mitochondrial respiration	positive impact on performance
	CKM	Playing a role in the phosphorylation paths of ATP and certain phosphagens	Enhancing the resistance to exhaustion (CKM‐knockout models)
	PDK4	Influencing glucose metabolism and regulates oxidation of fatty acids	positive impact on performance
	PPARs	Participating in regulations of the genes that impact fatty acids oxidation and glucose metabolism	Impact on exercise capacity
Phenotype modification	QTLs	Modulating, for example, weight and height	Achieving desired phenotype
	MSTN	Inhibiting the growth of skeletal muscles	Achieving muscle development by insertion of the SINE
	TBX15	Influencing skeletal development, skeletal muscle fibre differentiation	Achieving desired phenotype
Influencing disease susceptibility	MMP‐1	Breaking down the extracellular matrix^59^ Enhanced expression noted in OA, SDFT	Masking and decreasing injury susceptibility by blocking the expression
	PRG4	Providing the synovial fluid ability to disperse strain energy under the load^60^	Preventing the development and progression of OA
	IGF‐1	Influencing proteoglycan synthesis and enhancement in the type II collagen Creation	Masking and decreasing injury susceptibility
	IL1RN	expressing interleukin‐1 receptor antagonist protein, which acts as an inhibitor towards interleukin‐1	Faster recovery after the training Masking OA

Abbreviations: ACTN3, alpha‐actinin‐3; CKM, creatine kinase muscle; COX4I2, cytochrome c oxidase, subunit 4, isoform 2; EPO, erythropoietin; GH, growth hormone; HIF‐1, hypoxia‐inducible factor 1; IGF‐1, insulin‐like growth factor‐1; IL1RN, interleukin 1 receptor antagonist; MMP‐1, matrix metalloproteinase‐1; MSTN, myostatin; OA, osteoarthritis; PDK4, pyruvate dehydrogenase kinase, isoenzyme 4; PPARs, peroxisome proliferator‐activated receptors; PRG4, proteoglycan 4; QTLs, quantitative trait loci; SDFT, superficial digital flexor tendon; SINE, short interspersed nuclear elements; TBX15, T‐box transcription factor; VEGF, vascular endothelial growth factor.

*Source*: Racing NSW^60^ and Gruper.^61^

## 4 DETECTION METHODS

**FIGURE 2:**
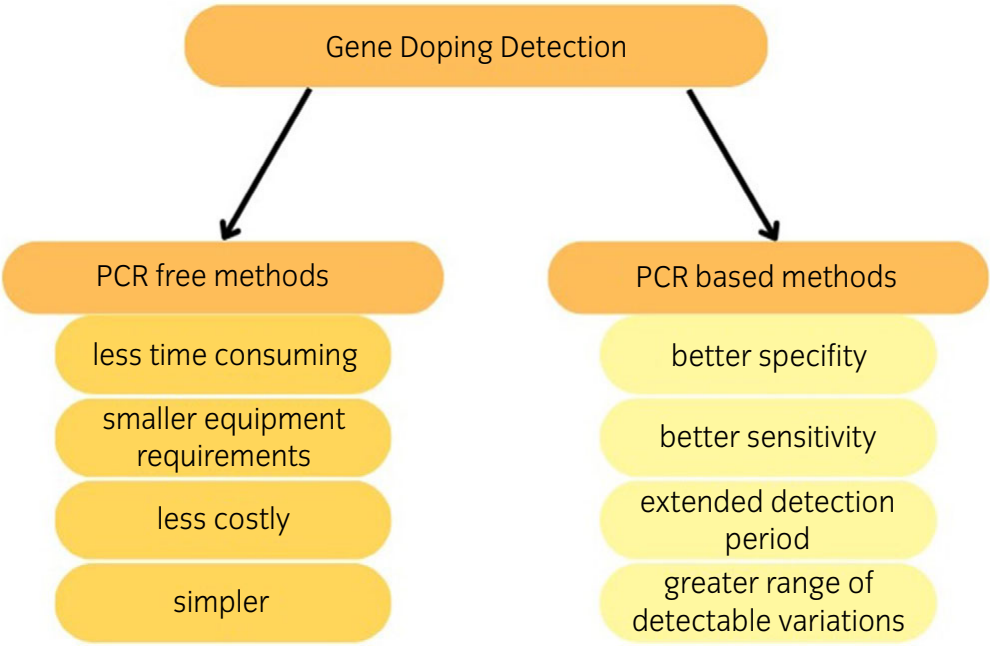
Comparison of the PCR‐free and based methods of gene doping detection.^1^, ^17^

Methods for detection and characterisation of gene therapy are summarised in Table 2 and there are two types of detecting methods: direct and indirect (see Figure 2).

**TABLE 2:** Gene therapy detection methods and their characteristics.

Detection method	Characterisation	Advantages	Challenges
Mass spectrometry	Identifies proteins and metabolites based on mass‐to‐charge ratio	Identifying several doping targets at the same time Significant scalability^23^	Probable reduced effectiveness after longer periods post administration^23^ Complex and expensive
UnLOCKing (SHERLOCK)	CRISPR‐based diagnostic tool	Does not require complex equipment Economical Great sensitivity and specificity Highly sensitive Multiplex testing Fast procedure^62^	Need high level of expertise to prepare and test reaction components^62^ Predesigned assays not commercially accessible
WGR	Whole genome resequencing for comprehensive analysis	Comprehensive coverage of an individual's entire genome^63^ High sensitivity (finding alterations without the known target)^64^	High cost^63^ Great number of data that needs to be interpreted, stored and analysed^63^ (computational resources needed)
DELLY method	Detects genetic rearrangements through split‐read and paired‐end algorithms	Effective for identifying genome modifications	Computational complexity
EBP	Equine biological passport for monitoring longitudinal changes in equine athletes	Long‐term monitoring of biological changes	Implementation and standardisation
PCR‐based methods	Based on the detection of the unique exon–exon junctions of the transgenes	Simple and economical Great sensitivity and specificity High precision and reliability^17^	Possibility of false positives because of pseudogenes, contamination and alteration in the junctions^65^ Small alterations in the transgene design could prevent PCR amplification^66^ Requirement of the complex equipment^62^ The absence of standardised protocols^62^
MFQPCR	Microfluidic Quantitative PCR	More economical than qPCR Easily adaptable by the creation of designated primers and probes Decreased amount of reagents^17^ High number of targets from many samples, simultaneously tested^17^	Limited flexibility in comparison to a qPCR^67^
Real‐time PCR	Amplifies DNA sequences to detect transgenes	High accuracy, specificity, sensitivity Flexibility (easy adjustment of the parameters of the reaction)^67^ Remotely economical Requires a remotely small amount of steps	Measurement of the absolute quantity of targeted sequences with the need of a calibration curve^22^ Need for higher amount of reagents^17^ Limited number of targeted genes
ddPCR	Digital PCR for detecting low copy numbers of transgenes	Enables direct measurement of the absolute quantity of targeted sequences without needing a calibration curve^22^ Not influenced by the efficiency of amplification^22^ Precision and sensitivity comparable/advantages over qPCR^66^ Greater resistance to PCR inhibitors in comparison with qPCR^66^ Detection with a great number of genomic DNA^68^	Hardship to differentiate false positive signals from positive ones in current digital PCR technologies^66^ Requires specialised equipment
Multiplex PCR	Simultaneously detects multiple transgenes in one reaction	Amplification of numerous targeted sequences Less time and funds consuming^56^	The need of optimisation (presence of nonspecific products due to a higher number of primers—impaired amplification, non‐desired interactions)^69^ The existence of preferential amplification (one designated sequence is biased in amplification)^69^

Abbreviations: ddPCR, droplet digital PCR; EBP, equine biological passport; MFQPCR, microfluidic quantitative PCR; WGR, whole genome resequencing.

### 4.1 Direct detection methods

Direct detection methods focus on PCR testing, whilst indirect methods rely on detecting the products of the gene intervention, such as proteins, RNA and metabolites. To detect such changes, the main challenge is establishing the nature of proteins, as it is problematic to distinguish endogenic molecules from those obtained from transgenes. When analysing potential transgenic proteins or metabolites, the literature suggests using ELISA or mass spectrometry.^7^ Identifying transgenes is somewhat easier due to their lack of intron regions in DNA. PCR methods are based on the detection of the unique exon–exon junctions of the transgenes. One of the challenges with this approach is the possible influence of pseudogenes within the host genome that can produce a false‐positive detection of a transgene.^65^

#### 4.1.1 Hair sample analysis

Tozaki et al.^18^ identified an opportunity for future gene doping detection tactics: they showed that tail hair can be used for genome screening. The main advantage of hair is that it can be preserved for around 10 years and is a great, practical sample. In addition, it seems like an attractive proposal for the control of genetically modified offspring, as any transformed genome would be detectable in the hair. Whole genome resequencing (WGR) can be also analysed from blood samples. However, there are undeniable advantages of the hair samples as they can be collected by anyone whereas blood samples should be collected by a veterinarian and are less easily transported. Hair samples are durable and facilitate storing data for a WGR library and for comparing the genome of a parent to its offspring for detection of gene editing in embryos.

#### 4.1.2 Detection of transgenes using microfluidic quantitative polymerase chain reaction

Tozaki et al.^21^ detected the 12 transgenes as possible targets of doping abuse using the MFQPCR (microfluidic quantitative polymerase chain reaction) method. The 12 genes, all discussed above, were *GH1*, *IGF1*, *EPO*, *MSTN*, *FST*, *ZFAT*, *FGF2*, *VEGF*, *CKM*, *PCK1*, *PDK4* and *PPARD*. This work established MFQPCR as an efficient way of screening for these genes and it is likely that this method could be adjusted for detecting other transgenes by creating new primers and probes.

#### 4.1.3 Droplet digital PCR technique

The droplet digital PCR (ddPCR) technique has been evaluated for the detection of gene doping in horses.^22^ This method was efficacious in detecting at least 130 and 200 copies of the EPO transgene in 1 mL plasma and urine, respectively. Potentially, the same procedure could be used for the identification of IGF1 and FST. The same results were obtained when EPO was built into a plasmid vector and ddPCR effectively detected the foreign gene from blood samples of infected micromini pigs (the study model).^70^

#### 4.1.4 Multiplex PCR gene detection

In a very recent study, multiplex PCR gene detection was shown to have excellent specificity and high sensitivity.^56^ Five designated transgenes were detected in one analysis.

#### 4.1.5 Confirmation test with PCR and sequencing

Combining PCR amplification and sequencing the DNA with primers and promoters indented to certain transgene identification makes it possible to rule out false positives.^65^ However, there are challenges in PCR methods and only a few known target genes can be detected in one reaction and small alterations in the transgene design could prevent PCR amplification.^71^

#### 4.1.6 Other PCR‐based methods

Maniego et al.^23^ used massively parallel sequencing through two‐step PCR to simultaneously target multiple transgenes with the combination of multiple sets of primers. Another interesting approach, discussed by Tozaki et al.,^72^ is split‐read and paired‐end algorithms in a method called DELLY, which detects genetic rearrangements and it has been proven that this method usefully detects certain transgenes. PCR methods are compared and summarised in Table 2.

### 4.2 Indirect detection methods

#### 4.2.1 Immunological response measurement

There are many possible tests for gene doping detection in humans.^17^ Measuring the body's immunological response to a viral vector could be one of them. However, horses are commonly exposed to adeno‐associated viruses with the resultant production of neutralising antibodies (NAbs) that could interfere with the immunological detection of a viral vector and consequently, this method has not been used in the detection of gene doping in horses.^7^

#### 4.2.2 Equine biological passport

Another indirect detection possibility relies on the concept of an equine biological passport (EBP). Longitudinal tracking of specific biological markers may reveal illicit genetic modifications over time by establishing baseline genetic profiles, conducting regular longitudinal tracking, employing advanced genomic analysis, and integrating data into a centralised database to monitor any changes that have occurred through, for example, gene doping.^7^, ^73^ Some racing authorities are already working on the development of the EBP.^60^

## 5 ETHICAL CONSIDERATIONS

People have been breeding horses to gain the best possible offspring for centuries. Is direct genetic alteration significantly different from what has been practised by selective breeding? There are serious ethical concerns around gene doping and its potential influence. Firstly, the accuracy of such methods is uncertain. For instance, the CRISPR technique can easily lead to inexact outcomes, such as off‐target effects that can cause damage or mutation of DNA with unknown impacts on the animal.^74^ Gene alteration has unknown potential impacts on individuals, some may be fortunate, others unfortunate.^75^ Failed integration of a transgene could provoke carcinogenesis.^12^ There are many factors influencing gene expression, including environmental modulators, potentially resulting in different outcomes of gene alteration in different individuals.^76^ Gene doping has a negative impact on the integrity of the sport and cheating that leads to the degradation of fair play and equestrian sports credibility.^77^ This would negatively impact the public's support of equestrian sport with potentially catastrophic financial consequences for the industry. In human sports, an athlete decides whether to participate or not, but an animal cannot give its consent, which, from an ethical point of view, is already problematic when it comes to the usage of animals for human entertainment, such as races. Natural breeding provides a balance, with some individuals being more suitable for competition than others, consequently allowing horses to exist in agreement with their abilities and predispositions.^66^

## 6 REGULATION AND ANTI‐DOPING EFFORTS

The IFHA has stated that: ‘Agents that are capable, at any time, of directly or indirectly causing an action or effect, or an action and effect on gene expression in any mammalian body. This includes but is not limited to gene editing agents with the capacity to alter genome sequences and/or the transcriptional, posttranscriptional or epigenetic regulation of gene expression.’^78^ Detecting such agents results in disqualification and repercussions for the personnel involved. IFHA have also declared that gene therapy is only acceptable with the acceptance of the Racing Authority for treatment of disease or injury diagnosed by a veterinarian, whilst fulfilling the following conditions: (a) ‘Not capable of modifying a horse's heritable genome’; (b) ‘Does not pose a threat to the welfare of horse’; (c) ‘Does not pose a threat to the integrity of racing, either by having the potential to enhance or harm the performance of a horse in a race.’^78^ In 2017, the IFHA formed the Gene Doping Control Sub‐committee to define gene therapy and its misuse in the racing industry. At the current time, IFHA has made significant strides in addressing gene doping but has not yet established specific detection criteria.

Meanwhile, WADA published ‘Guidelines on Gene Doping Detection’ in 2021, stating that direct PCR‐based analytical methods are the laboratory method of choice.^71^ The authorities all across the world recognised the importance of harmonisation and regulation in developing anti‐doping strategies. Consequently, in 2012, IFHA described one of the main goals for the years 2012–2015 as: ‘harmonisation of rules and harmonisation of medication control’.^79^ In the objectives for 2020–2023, it was stated that one of the areas of focus will be cooperation with the Gene Doping Control Sub‐committee to enhance the control and detection of gene doping. Official collaboration with WADA was established in 2019, which was highly recommended by the anti‐doping advocates.^79^, ^80^

The cooperation of regulatory bodies, veterinarians, scientists, and stakeholders is crucial in developing proper strategies to combat gene doping. As regulatory bodies are conducting frameworks and guidelines, they need to collaborate with scientists who are working among others on developing the most accurate detection methods. An example of such a project was the 2019 AORC Gene Doping Workshop, during which gene therapy and gene doping were discussed by scientists, veterinarians, doctors, and world leaders from 12 countries.^80^ Such initiatives ensure collective action towards combating the new challenges within the sports community.

## 7 PUBLIC PERCEPTION AND STAKEHOLDER ENGAGEMENT

The public's opinion on horse doping was evaluated in an online survey^81^ showing that 53.5% of correspondents were concerned about the potential usage of gene doping in horse racing, viewing it as a threat to equine welfare. The survey indicated that people value both the ethics of the sport and care for the welfare of the animals. Seven of 10 of the younger survey participants were anxious about the risks to the health of equine athletes. The survey results suggest that around 46% of respondents would be more eager to follow horse races if there was more ethical transparency. Nearly 83% of younger respondents expressed their doubts concerning the ethics of horse racing and hesitancy to support racing as a result. In recent years, there has been a decrease in horse racing interest, thus enhancing ethical transparency would be helpful for the industry and the survey highlighted the importance of fairness, communication and ethics in horse racing.^81^ The future of equestrian sport is heavily reliant on the Social Licence to Operate^82^ and the engagement of a public which prioritises the welfare of equestrian athletes and transparency.^83^, ^84^ In a UK survey conducted, only 18% of the respondents answered that if communication and welfare stay at the current level, horse racing will continue to be acceptable.^59^

## 8 FUTURE PERSPECTIVES

At the current time, most reports of gene therapy in horses are in experimental studies, although it has potential clinical applications.^7^ Currently, in human medicine, there are 22 approved in vivo and ex vivo products for gene therapy. Major future challenges of such therapies relate to their safety, effectiveness, and specificity. Their issues concerning the choice of vectors; some trigger troublesome immune responses, whilst others are not efficient. Regulatory and ethical concerns that surround potential promising treatments remain to be addressed.^85^

Work on gene doping detection is progressing rapidly: Lu et al.^17^ developed a CRISPR/Cas‐based method to assess gene doping corresponding with a method proposed by Sung's group, which uses CRISPR/deadCas9 for the detection of EPO gene doping in humans. Although it has beneficial applications, the CRISPR method could also be used to enhance athletic performance capacities.^17^ The Specific High Sensitivity Enzymatic Reporter UnLOCKing (SHERLOCK) approach has potential as a test for doping control samples in the future.^86^ Ohnuma et al.^87^ proposed an innovative approach to gene‐doping detection, applying the πCode method capable of multiplex detection, identifying many biomarkers in one diagnostic test.

As of now, there have been no publicly confirmed positive cases of gene doping in sport horses and the prevalence of gene doping practices in equestrian sports is still unknown. The relatively recent development and implementation of advanced gene doping detection methods may mean that comprehensive testing has not been widespread enough to identify any cases of misuse. However, sporting authorities and scientists are working intensively on establishing new and accurate detection methods to identify gene doping. Ethical debates are taking place^61^, ^88^ calling for cohesiveness and unity in sports communities that will allow the creation of accurate guidelines.

## 9 ANTI‐GENE DOPING STRATEGIES IN HORSE SPORTS

**FIGURE 3:**
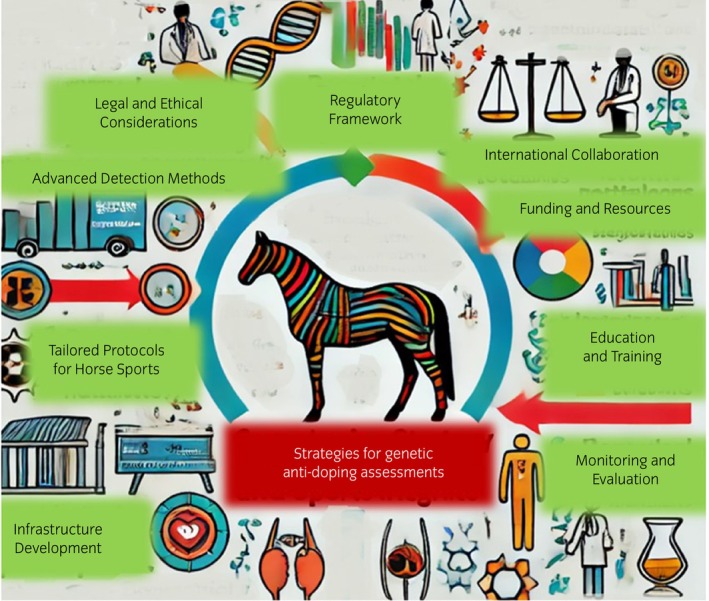
Possible strategies for genetic anti‐doping assessments.

Implementing anti‐gene doping assessment in horse sports across different jurisdictions requires a coordinated effort involving regulatory alignment, infrastructure development, specialised training, advanced detection methods, international collaboration, and ethical considerations. By leveraging these strategies, it is possible to enhance the integrity of horse racing and equine sports and ensure fair competition globally. Figure 3 illustrates the possible strategies for implementing anti‐gene doping assessment in different jurisdictions and horse sports.

### 9.1 Regulatory framework

A robust regulatory framework is essential for the successful implementation of anti‐gene doping assessments. This involves adopting international standards, national legislation and compliance monitoring.^12^ Countries should align with the guidelines set by the IFHA,^89^ the FEI^3^, ^18^ and the WADA^12^ to ensure consistency and credibility across different jurisdictions. In addition, the integration of gene doping regulations within national anti‐doping laws provides a legal foundation for enforcement and sanctions. National governments must support these measures through appropriate legislation. Mechanisms should be established to monitor and ensure compliance with these regulations at both national and international levels. This ensures that the rules are uniformly applied and adhered to.

### 9.2 Infrastructure development

Developing the necessary infrastructure is key to conducting effective anti‐gene doping assessments by regional testing centres, accreditation and standards and mobile genomic units. Establishing regional laboratories equipped with advanced genomic analysis technology can reduce dependency on a few large centres. These laboratories should be strategically located to cover wide geographic areas and ensure accessibility. In addition, regional centres must be accredited and adhere to international standards for testing and quality control to maintain credibility and reliability. Also, deploying mobile units capable of collecting and processing genetic samples allows for on‐site testing at various competitions and events, including those in remote and smaller venues.

### 9.3 Tailored protocols for horse sports

Each equine discipline has unique requirements that must be considered in the development of anti‐doping protocols by discipline‐specific guidelines, breed‐specific considerations and periodic reviews and updates. Anti‐doping protocols should be tailored to different equine disciplines recognising their unique demands and risk factors. Testing protocols should also account for genetic differences among various horse breeds to ensure accurate and fair assessments.^18^ Regularly reviewing and updating these protocols based on new scientific findings and technological advancements is essential to stay ahead of doping methods.

### 9.4 Education and training

Education and training are critical in the fight against gene doping.^12^ Specialised training should be provided for veterinarians and laboratory technicians on the latest gene doping detection techniques and ethical sample collection methods. Also, educational programs for owners, trainers, riders and other stakeholders can raise awareness about it, its risks, and the importance of genetic integrity in horses.^59^ As well as conducting campaigns to educate the broader public, it helps garner support for anti‐doping initiatives and promotes a culture of fair play in equine sports.

### 9.5 Advanced detection methods

Investing in advanced detection methods to identify gene doping is critical. Cutting‐edge technologies like CRISPR screening,^17^, ^62^, ^86^ whole genome sequencing^6^, ^17^, ^63^, ^64^ and epigenetic markers^23^, ^68^, ^69^, ^90^ all show potential. Implementing genetic, biological passports that track the genetic profiles of horses over time can help detect anomalies indicative of gene doping.^7^, ^73^ Continuous research and development efforts will be necessary to improve existing methods and develop new techniques.^6^

### 9.6 International collaboration

International collaboration is vital for the global fight against sport horse doping.^71^, ^79^ Establishing partnerships between countries allows for the sharing of resources, expertise, and best practices in gene doping detection. Utilising and contributing to international databases that track genetic profiles and doping test results enhances global oversight and intelligence sharing.^59^, ^71^

### 9.7 Funding and resources

Securing adequate funding and resources is crucial to fund anti‐doping programs, including research, infrastructure, and training efforts to attract sponsorship from many sources such as research grant agencies, private sector entities, including businesses and philanthropic organisations are important. Developing cost‐effective genomic testing methods ensures that smaller and less wealthy jurisdictions can also participate in effective anti‐doping assessments.

### 9.8 Legal and ethical considerations

Legal and ethical considerations must be at the forefront of anti‐doping measures.^71^ Prioritising the welfare of horses in all genetic testing methods ensures that procedures are non‐invasive and do not harm the animals. Maintaining high ethical standards in the conduct of some tests, including transparency, confidentiality, and fairness, is essential.^79^

### 9.9 Monitoring and evaluation

Continuous monitoring and evaluation help maintain the effectiveness of anti‐doping measures.^71^ Regularly reviewing and updating anti‐doping policies based on the latest scientific and technological developments is crucial. Conducting independent audits of anti‐doping programs ensures compliance with international standards, identifies areas for improvement, and maintains credibility. Establishing metrics to evaluate the effectiveness of anti‐doping strategies and making data‐driven adjustments as needed helps maintain the integrity of the program.

## 10 CONCLUSIONS

The ongoing evolution of genetic technologies offers promising solutions but also threats. Genes that could be the target of doping as they alter the athletic phenotype and capacities have been identifed. Regulatory bodies have recognised this as a real threat while facing the challenge of developing official detection guidelines. Only a collective attempt of veterinarians, authorities, and researchers will ensure the welfare of horses and effective regulatory frameworks and maintain public trust and support of equestrian sports. Prioritisation of equine welfare and transparency is what the public expects and these efforts are also crucial for preserving the spirit of fair competition in equine sports.

## FUNDING INFORMATION

The publication was financed by Narodowe Centrum Nauki (2021/41/B/NZ7/03548) and Science development fund of the Warsaw University of Life Sciences—SGGW.

## CONFLICT OF INTEREST STATEMENT

The authors have declared no conflicting interests.

## AUTHOR CONTRIBUTIONS

**Maria Puchalska:** Writing – original draft; writing – review and editing; investigation. **Olga Witkowska‐Piłaszewicz:** Conceptualization; writing – original draft; writing – review and editing; funding acquisition; investigation; supervision.

## DATA INTEGRITY STATEMENT

Not applicable.

## ETHICAL ANIMAL RESEARCH

Not applicable.

## INFORMED CONSENT

Not applicable.

## Data Availability

Data sharing is not applicable to this article as no new data were created or analysed in this study.
